# Reduction in low-dose to normal tissue with the addition of deep inspiration breath hold (DIBH) to volumetric modulated arc therapy (VMAT) in breast cancer patients with implant reconstruction receiving regional nodal irradiation

**DOI:** 10.1186/s13014-018-1132-9

**Published:** 2018-09-24

**Authors:** Vishruta A. Dumane, Kitwadee Saksornchai, Ying Zhou, Linda Hong, Simon Powell, Alice Y. Ho

**Affiliations:** 1 Department of Radiation Oncology, Icahn School of Medicine at Mount Sinai, 1184 5th Avenue, Box 1236, New York, NY 10029 USA; 2 Department of Medical Physics, Memorial Sloan Kettering Cancer Center, 1275 York Avenue, New York, NY 10065 USA; 3 Department of Radiation Oncology, Memorial Sloan Kettering Cancer Center, New York, NY USA

**Keywords:** VMAT, DIBH, IMRT, Tissue expanders, Permanent implants, Cardiac dose, Low dose

## Abstract

**Background:**

Despite dosimetric benefits of volumetric modulated arc therapy (VMAT) in breast cancer patients with implant reconstruction receiving regional nodal irradiation (RNI), low dose to the thoracic structures remains a concern. Our goal was to report dosimetric effects of adding deep inspiration breath hold (DIBH) to VMAT in left-sided breast cancer patients with tissue expander (TE)/permanent implant (PI) reconstruction receiving RNI.

**Methods:**

Ten consecutive breast cancer patients with unilateral or bilateral TE/PI reconstruction who were treated with a combination of VMAT and DIBH to the left reconstructed chest wall and regional nodes were prospectively identified. Free breathing (FB) and DIBH CT scans were acquired for each patient. VMAT plans for the same arc geometry were compared for FB versus DIBH. Prescription dose was 50 Gy in 25 fractions. Dosimetric differences were tested for statistical significance.

**Results:**

For comparable coverage and target dose homogeneity, the mean dose to the heart reduced on average by 2.9 Gy (8.2 to 5.3 Gy), with the addition of DIBH (*p* < 0.05). The maximum dose to the left anterior descending (LAD) artery was reduced by 9.9 Gy (*p* < 0.05), which related closely to the reduction in the maximum heart dose (9.4 Gy). V05 Gy to the heart, ipsilateral lung, contralateral lung and total lung (*p* < 0.05) decreased on average by 29.6%, 5.8%, 15.4% and 10.8% respectively. No significant differences were seen in the ipsilateral lung V20 Gy or mean dose as well as in the mean contralateral breast/implant dose. However, V04 Gy and V03 Gy of the contralateral breast/implant were respectively reduced by 13.2% and 18.3% using DIBH (*p* < 0.05).

**Conclusion:**

Combination of VMAT and DIBH showed significant dosimetric gains for low dose to the heart, lungs and contralateral breast/implant. Not surprisingly, the mean and maximum dose to the heart and to the LAD were also reduced. DIBH should be considered with the use of VMAT in breast cancer patients with implant reconstructions receiving RNI.

## Background

Immediate breast reconstruction offers significant quality-of-life benefits in women with breast cancer and is increasingly being used in the setting of radiation [1–4]. Regional nodal irradiation (RNI) is commonly administered to these patients. Owing to the anatomic challenges of targeting the internal mammary nodes in women with prosthetic reconstructions, conventional techniques in the setting of immediate reconstruction can significantly increase the dose to the heart and lungs, or fail to obtain adequate coverage of target volumes, compared to non-reconstructed patients receiving RT [5, 6]. Treatment planning techniques such as VMAT and multibeam IMRT have been exploited over the past decade to improve cardiopulmonary sparing for patients with immediate breast reconstruction. Both techniques have been shown to significantly improve sparing of the heart and lungs while generating conformal and homogeneous dose distributions to the target in breast cancer patients [7–9]. However, the resultant low dose exposure to normal organs with VMAT argues against the more widespread use of this technique in early stage breast cancer patients. The unique advantage of VMAT over multibeam IMRT is that it requires fewer monitor units (MU) and shorter delivery time, therefore enabling its combination with deep-inspiratory breath hold (DIBH) techniques that may further minimize dose to the heart. The aim of our study here is to report on the dosimetric effects of adding DIBH to breast cancer patients with implant reconstruction treated with VMAT.

## Methods

### Patient selection

As part of an IRB-approved protocol at Memorial Sloan-Kettering Cancer Center assessing the efficacy of VMAT for the treatment of breast cancer patients with reconstruction(s), we prospectively identified 10 consecutive breast cancer patients who were treated with a combination of VMAT and DIBH to the left reconstructed breast/chest wall and regional nodes (RN). It was routine practice to offer both a free-breathing and breath-hold scan for patients with left-sided breast cancer in whom regional nodal irradiation was planned and could reproducibly hold their breath during simulation. Both scans were performed in the same simulation session. For this study population (left-sided breast cancer patients with implant-based reconstruction requiring RNI), additional dose from the breath-hold scan was considered low relative to the dosimetric benefits that could potentially be achieved by planning on the breath hold scan compared to the free breathing scan. These patients are otherwise at risk for high lung and heart doses along with suboptimal target coverage when standard 3D-conformal techniques are used. Hence any additional dose from the breath-hold CT scan, as well as an apparent increase in complexity of treatment delivery as a result of combining DIBH with VMAT seems justified in these node-positive post-mastectomy patients with reconstruction. All patients had stage II-III breast cancer and had undergone mastectomy and immediate tissue expander (TE) placement. Among them, 4 had permanent implant (PI) and 6 had TE. Five patients had bilateral implants or TE. Two CT scans, one free breathing (FB) and one DIBH were acquired per patient, the latter of which were acquired using the Real-Time Position Management (RPM) respiratory gating system (Varian Medical Systems) using breathing instructions. CT scans were acquired at 3 mm slice spacing.

### Target delineation

The clinical target volume (CTV) consisted of the chest wall, implant, overlying skin, level I-II axillary lymph nodes, supraclavicular nodes, level III nodes and the internal mammary nodes (IMNs), which were included in the first 4 intercostal spaces. The planning target volume (PTV) was CTV + 5 mm and included the skin in the reconstructed breast/chest wall region. This margin was provided to account for respiratory motion and setup errors. The chest wall and lymph nodes were contoured as per published guidelines [10]. Both lungs were defined using the autocontour function in Eclipse Version11. The silhouette of the heart was contoured from the aortic arch superiorly extending inferiorly to the left ventricle. The contralateral implant was defined as the contralateral prosthesis including the skin. Other structures contoured were the contralateral breast (for unilateral cases) left anterior descending (LAD) artery, thyroid, esophagus and brachial plexus.

### VMAT planning

VMAT plans generated in this study followed a previously reported technique [9]. The angle at which the largest separation of the PTV is projected in the beam’s eye view (BEV) is chosen. This separation is typically found to be > 15 cm and due to limitations on the MLC leaf travel within an individual field (which is 15 cm on a VARIAN LINAC), a minimum of two complementary coplanar arcs were used to cover the PTV. The two coplanar arcs had a 2 cm overlap by the isocenter and the collimator angle used was 0°. The range of each arc was 190°-220° and both arcs were optimized simultaneously. Energy used for planning was 6MV. Planning was performed on both the FB and DIBH scans. The optimization algorithm used was the Progressive Resolution Optimizer (PRO) and the dose calculation algorithm was Analytical Anisotropic Algorithm (AAA), both V11. Prescription dose was 50 Gy in 25 fractions. No skin flash or virtual bolus was used during optimization. A 3 mm bolus over the chest wall was used on a daily basis. No patients received a chest wall boost.

### Planning objectives

For each case, the plan was optimized such that constraints for PTV coverage were met while those for all the critical organs were not violated as in Table 1. Priority was given to cover 95% of the IMNs with at least 100% of the prescription dose while achieving PTV D95, V95 ≥ 95% and PTV D05 ≤ 110%, followed by mean heart dose (MHD), ipsilateral lung V20 Gy and dose to the contralateral breast. Plans were normalized such that 95% of the target volume received 95% of the prescription dose while noting values of V95, dose inhomogeneity (D05) and doses to organs at risk (OAR). While planning these cases, our priority was to cover the IMNs such that the D95 ≥ 100% while maintaining the MHD ≤ 9 Gy. However if it was not possible to fulfill this criterion while still maintaining the MHD ≤ 9 Gy, we then attempted D95 of the IMNs to be ≥90%, while accepting a lower MHD at ≤8 Gy. The plans were optimized such that the criteria in Table 1 were met.

**Table 1:** Dosimetric planning guidelines for breast VMAT

Structure	Parameter	Objective
PTV	D95 (%)	≥95%
	V95 (%)	≥95%
	D05 (%)	≤110%
IMN	D95 (%)	≥100%
Ipsilateral Lung	V20 Gy (%)	≤33%
	V10 Gy (%)	≤68%
	Mean (Gy)	≤20Gy
Contralateral Lung	V20 Gy (%)	≤8%
Heart	V25 Gy	≤25%
	Mean (Gy)	≤9Gy^a^; ≤8Gy^b^
	Dmax (Gy)	≤50Gy
LAD	Dmax (Gy)	≤50Gy
Contralateral intact breast	Mean (Gy)	≤5Gy
Contralateral implant	Mean (Gy)	≤8Gy
Esophagus	Dmax (Gy)	≤50Gy
Thyroid	Mean (Gy)	≤20Gy
Brachial Plexus	Dmax (Gy)	≤55Gy

^a^If IMN D95 ≥ 100%

^b^If IMN D95 ≥ 90%

### Dosimetric evaluation

Dose volume histograms (DVH) were generated for PTV and critical organs indicated in Table 1. Dosimetric parameters collected for plans on FB and DIBH scans for each patient were compared. Statistical analysis was performed in MATLAB using the Wilcoxon signed-rank test for paired data at a significance level of ≤0.05. This test is a non-parametric hypothesis test used when comparing two related samples and does not assume the population to be normally distributed.

## Results

**Fig. 1:**
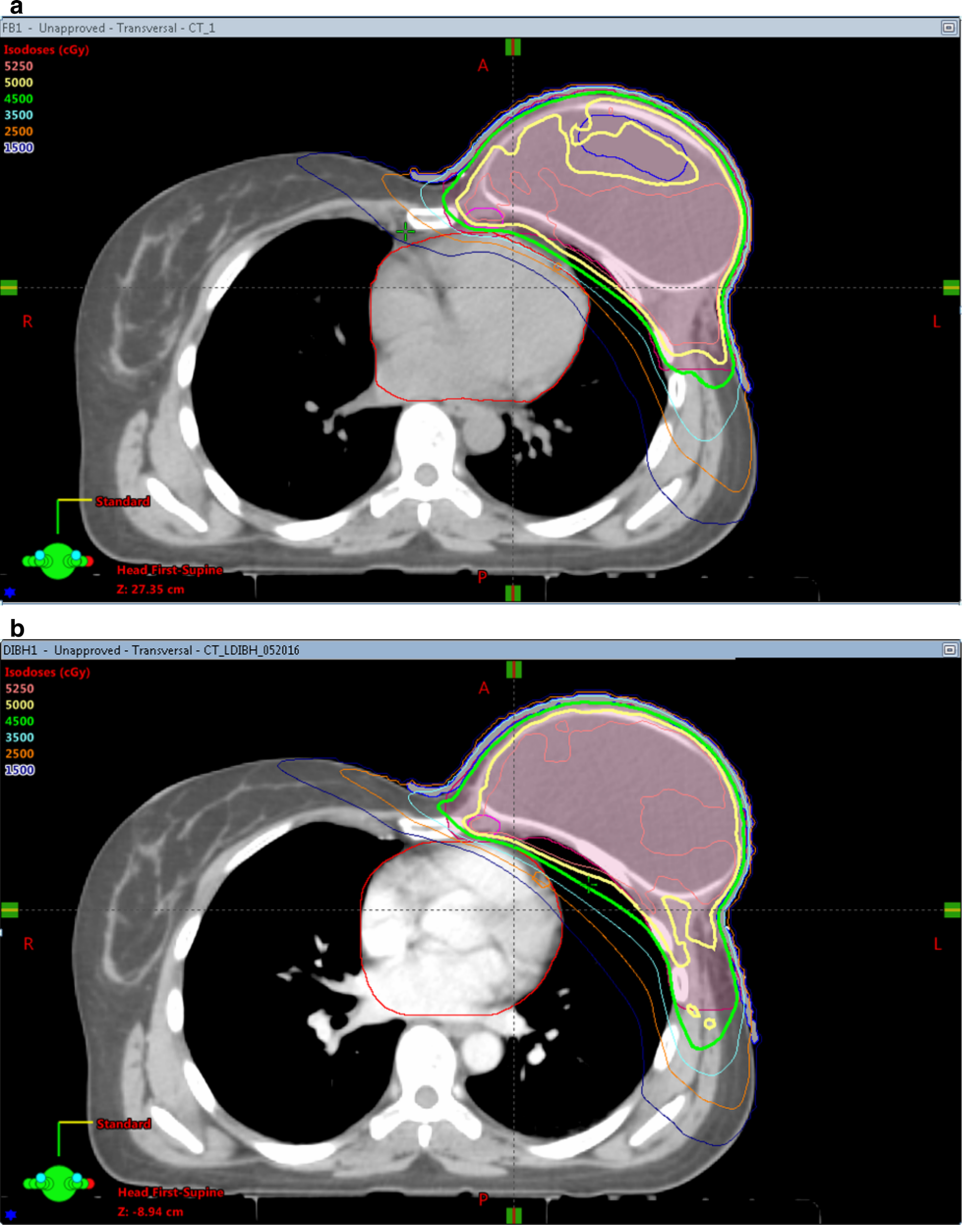
**a** Dose distribution in the axial plane for a free breathing plan. **b** Dose distribution in the corresponding axial plane for a DIBH plan

Dosimetric goals have been shown in Table 1. Values of the dosimetric parameters averaged over 10 patients for FB plans versus DIBH plans are shown in Table 2. Comparison of the dose distribution for the two treatments is shown in Fig. 1.

**Table 2:** Dosimetric comparison between coverage and OAR doses with DIBH versus FB

Structure	Parameter	FB	DIBH	*p* value
PTV	D95 (%)	95	95	–
	V95 (%)	95	95	–
	D05 (%)	111.9 ± 2.2	110.4 ± 0.8	0.07
IMN	D95 (%)	99.3 ± 4.2	100.9 ± 0.8	0.48
Ipsilateral Lung	V20 Gy (%)	28.2 ± 4.7	26.4 ± 3.9	0.1
	V10 Gy (%)	47.1 ± 6.9	44.7 ± 6.4	0.03
	V05 Gy (%)	77.2 ± 8	71.4 ± 7.5	0.01
	Mean (Gy)	15.7 ± 1.8	14.9 ± 1.6	0.03
Contralateral Lung	V20 Gy (%)	0.9 ± 1.5	0.7 ± 0.9	0.63
	V05 Gy (%)	48.5 ± 12.9	33.1 ± 18.3	< 0.01
Total Lung	V20 Gy (%)	13.1 ± 2.6	12.4 ± 2.1	0.12
	V10 Gy (%)	28.6 ± 6.1	24.6 ± 5.4	0.01
	V05 Gy (%)	61.3 ± 9.7	50.5 ± 12.2	< 0.01
	Mean (Gy)	10.3 ± 1.3	9.2 ± 1.4	< 0.01
Heart	V25 Gy (%)	3.1 ± 2.1	1 ± 1.5	< 0.01
	V15 Gy (%)	10 ± 4.4	2.9 ± 2.9	< 0.01
	V05 Gy (%)	64.9 ± 14.2	35.3 ± 9.9	< 0.01
	Mean (Gy)	8.2 ± 1.4	5.3 ± 1	< 0.01
	Dmax (Gy)	47.7 ± 7.5	38.3 ± 11.2	< 0.01
LAD	Dmax (Gy)	40.7 ± 12.1	30.8 ± 12.6	0.02
Contralateral Breast/Implant	V04 Gy (%)	48.5 ± 15.2	35.3 ± 12.9	< 0.01
	V03 Gy (%)	67.3 ± 16.8	49 ± 14.8	< 0.01
	Mean (Gy)	5.7 ± 1.4	5.1 ± 1.4	0.16
Esophagus	Dmax (Gy)	35.1 ± 8	33.1 ± 11	0.56
Thyroid	Mean (Gy)	13.4 ± 1.4	13.9 ± 2.1	0.87
Brachial Plexus	Dmax (Gy)	51 ± 1.1	51.5 ± 1.3	0.29

### PTV coverage

No significant differences in coverage of the PTV and IMNs was seen between planning on FB versus DIBH scans. Both coverage and homogeneity were met within ±1% of the dosimetric goals in Table 1.

### Heart and LAD

Compared to FB, with DIBH, the mean dose to the heart was reduced on average by 2.9 Gy and the maximum dose to the same was reduced by 9.4 Gy. The heart V25 Gy was reduced only by 2%. However, volume of the heart covered by low doses such as 15 Gy and 5 Gy was reduced by 7.1% and 29.6% respectively and the maximum dose to the LAD was reduced by 9.9 Gy with DIBH. All decreases in the heart dose were found to be statistically significant.

### Lungs

Compared to FB, with DIBH, no statistically significant differences were seen in the ipsilateral lung V20 Gy, while the ipsilateral lung mean dose differed by < 1%. Volumes of the ipsilateral lung covered by lower doses namely the V10 Gy and V05 Gy were reduced by 2.4% and 5.8%, both of which were statistically significant. The V20 Gy to the contralateral lung showed no significant difference but, the contralateral lung V05 Gy was reduced by 15.4%, a result found to be statistically significant. A comparison of doses to the total lung showed that the greatest advantage of using DIBH was seen in the low dose, namely the V05 Gy, which was reduced by 10.8%.

### Contralateral breast

No significant differences were seen in the mean dose to the contralateral breast/implant; however, V04 Gy and V03 Gy were reduced by 13.2% and 18.3% respectively with DIBH compared with FB.

### Other structures

Doses to the esophagus, brachial plexus, and thyroid showed no significant differences between FB versus DIBH plans.

## Discussion

Several trends in the treatment of breast cancer patients over the past decade have directly impacted RT planning priorities. Primarily, rates of immediate reconstruction have dramatically risen over the past decade, owing largely to the enhanced use of implants [11]. Second, indications for RNI in 1–3 node positive breast cancer patients were expanded following the publication of two randomized trials that showed a small but statistically significant benefit in disease-free survival in patients who received RNI [12, 13]. Although the vast majority of the patients on these two trials were largely treated with breast-conserving therapy, results have been extrapolated to women receiving mastectomy, raising new questions about how to optimize RT planning when RNI is required. Finally, a large population-based study conducted in Swedish and Danish patients demonstrated a dose-response relationship between mean heart dose and the development of major cardiac events in breast cancer patients treated with radiation [14]. Collectively, these developments underscore the increasing importance of novel but pragmatic treatment planning approaches that are designed to cover the target volume while minimizing dose to the heart and lung. With this in mind, we performed a dosimetric comparison of FB versus DIBH plans for patients with left-sided breast cancer with immediate implant based reconstruction receiving RNI with VMAT. The main findings of our study were that the addition of DIBH to VMAT helps reduce volume of the heart, lungs and contralateral breast/implant covered by a range of doses. In addition, the combination of DIBH with VMAT also decreased mean heart dose as well as maximum doses to heart and LAD, compared to FB. The reduction in mean heart dose would be expected to have a significant impact on late cardiac events, based on dose effect curves found by Darby et al.

Conventional 3D conformal planning with tangential photon beam arrangement in patients requiring RNI can be challenging in the setting of reconstruction [5]. Treatment planning challenges are particularly magnified in patients with bilateral reconstructions. In a dosimetric analysis of 197 patients with implant reconstructions, of whom 49% had bilateral implants, irradiation of the IMNs was found to be an independent predictor for increased dose to the heart, lungs and the contralateral implant [6]. Ohri et al. showed that, the average ipsilateral lung V20 Gy was 23.8% when IMNs were not included in the target volume compared to 36.9% when they needed to be covered in presence of an implant reconstruction. Likewise, the MHD was 3.3 Gy versus 6 Gy respectively. Results from these studies indicate that when radiation to the IMNs is required, standard 3D conformal planning techniques may not give sufficient coverage to the IMNs and more advanced radiotherapy planning and delivery techniques such as multibeam IMRT or VMAT may be required. We have previously reported on the feasibility of multibeam IMRT for patients who receive postmastectomy radiation therapy (PMRT) to the TE/PI and regional lymph nodes [15]. The main advantage of VMAT over multibeam IMRT with static fields is reduced MU and a quicker delivery time to produce comparable or even improved sparing of the OARs while achieving the desired target coverage. Regardless of whether multibeam IMRT or VMAT is used, the phenomenon of increased low dose to normal tissue is a common concern. Combining VMAT and DIBH takes advantages of both techniques to improve normal tissue sparing without compromising the desired target coverage.

Previous studies comparing 3D conformal planning versus VMAT +/− DIBH have been performed in breast conserved patients [16]. Osman et al. reported that the combination of VMAT and DIBH led to an increase in MHD compared to tangential beam arrangements with DIBH except when the MHD with the latter was > 3.2 Gy. A similar study performed by Pham et al. [17] compared the use of tangential IMRT and VMAT +/− DIBH. They reached a similar conclusion that VMAT with DIBH only helped to reduce the MHD if it was > 6.3 Gy when using a combination of tangential field IMRT with DIBH. In our study, tangential field arrangements were not a feasible option given the necessity of covering the IMNs and adjacent medial contralateral prosthesis. VMAT is likely to be a better option in this situation, and our results indicate that the addition of DIBH will be of dosimetric benefit for these cases.

Quantitative analyses of normal tissue effects in the clinic (QUANTEC) recommends the heart V25 Gy be kept under 10% at a fractionation of 2 Gy in order to minimize the probability of death from a cardiac event at 15 years post RT to be < 1% [18]. Due to VMAT’s capability of delivering with a continuous arc covering all angles, it is possible to better carve out high dose areas (≥ 20 Gy) around the heart compared with tangential beam arrangements [8, 9, 17]. The heart V25 Gy noted in our study was well below 10% with FB (at 3.1%) and DIBH (at 1%). The influence of low dose, specifically the volume of the heart covered by 1 to 2 Gy isodose line on heart disease has been investigated at the University of Michigan in breast cancer patients treated with standard tangential fields [19]. No correlation was found between low dose and cardiac function or perfusion defects, and no worsening of these defects occurred within a short-term follow-up (1 year) after RT. While encouraging, these results cannot be extrapolated to VMAT, which results in a different distribution of low-dose spread through the use of arcs. Given the absence of data on the long-term cardiac effects in breast cancer patients treated with multibeam IMRT or VMAT, conservative goals dictate heart doses as low as can be achieved while maintaining adequate coverage to the target. In our study, the MHD on average with FB was 8.2 Gy (5.6 Gy – 9.7 Gy) and was reduced to 5.3 Gy (4.1 Gy – 6.6 Gy) with the addition of DIBH. The heart V5 Gy was reduced on average by 29.6% with DIBH. Although the significance of a large volume of heart receiving a low dose of radiation is currently unknown, studies have found an association between high dose regions in the heart from RT to the breast and radiation-induced microvasculature injury such as the LAD, which can contribute to the cardiac mortality after RT [20]. We found that with DIBH, the maximum dose to the heart and the LAD were both reduced by almost 10 Gy compared to FB.

The ipsilateral lung V20 Gy was < 30% with both FB and DIBH, but slightly improved with the DIBH. The mean dose to the ipsilateral lung and total lung were comparable between the two techniques. These observations are in agreement with previously published dosimetric studies comparing the two techniques [16, 17]. It is well known that the risk of RT-induced lung morbidity is influenced by the total dose, dose per fraction and the volume of irradiated lung [21, 22]. One of the caveats of VMAT is that it increases the volume of irradiated lung exposed to low dose. In our study, we found that by adding DIBH to VMAT, the V05 of the ipsilateral and contralateral lung was decreased by 11% and 15.4% respectively. There is some suggestion that V05 Gy of the contralateral lung is an important predictor of radiation pneumonitis (RP) for patients receiving concurrent chemotherapy for esophageal cancer [23]. The study reported that contralateral lung V05 Gy of 58% or more was associated with symptomatic RP (≥ grade 2). In our study, the contralateral lung V05 Gy was on average 33.1% with DIBH and 48.5% with FB. Three patients were found to have contralateral lung V5 Gy > 60%, which was reduced with DIBH to < 50%.

Dose to the contralateral breast from radiotherapy and risk of second primary breast cancer was examined in the women’s environmental cancer and radiation epidemiology (WECARE) study. In this study, very young patients (defined as < 40 years of age) who received dose > 1 Gy to the contralateral breast were at a higher risk of developing contralateral breast cancer [24]. More importantly, there was a direct correlation between dose and risk, making the avoidance of low dose exposure to the contralateral breast an important goal of radiation treatment planning. However, when standard tangential beams are used, especially in patients with bilateral TE/PI, a significant portion of the contralateral breast/implant gets full dose in order to adequately cover the target and IMNs; as a result, the average maximum dose to the contralateral breast/implant is almost 80% of the prescription dose [6]. Although the low dose bath is still typically higher with VMAT, our study has shown that this can be significantly mitigated with DIBH. Even though the mean doses to the contralateral breast/implant are similar (28%) with FB versus DIBH, the volume of the contralateral breast/implant covered by 3 Gy and 4 Gy was reduced by 18.3% and 13.2% respectively with DIBH over FB. Due to chest wall expansion with DIBH, the separation between the ipsilateral and contralateral implant is increased, helping to reduce volume of the contralateral implant exposed to low dose. Maximum doses to the esophagus and brachial plexus as well as the thyroid mean dose showed minimal to no difference between FB versus DIBH. This was because chest wall expansion did not increase the distance between the esophagus and the thyroid from the PTV, while the brachial plexus on both FB and DIBH scans was a part of the PTV.

## Conclusions

By reducing low-dose exposure for the heart, lungs and contralateral breast by an average of 29.6%, 10.8% and 18.3%, respectively, the addition of DIBH to VMAT is a practical and valuable RT planning strategy for breast cancer patients with implant reconstruction requiring RNI.

## Abbreviations

AAA: Analytical Anisotropic Algorithm
BEV: Beam’s eye view
CTV: Clinical target volume
DIBH: Deep inspiration breath hold
DVH: Dose volume histogram
FB: Free breathing
IMN: Internal mammary node
IMRT: Intensity modulated radiation therapy
LAD: Left anterior descending
MHD: Mean heart dose
MU: Monitor unit
OAR: Organ at risk
PI: Permanent implant
PMRT: Postmastectomy radiation therapy
PRO: Progressive Resolution Optimizer
PTV: Planning target volume
QUANTEC: Quantitative analyses of normal tissue effects in the clinic
RNI: Regional nodal irradiation
RP: Radiation pneumonitis
RPM: Real-Time Position Management
TE: Tissue expander
VMAT: Volumetric modulated arc therapy
WECARE: Women’s environmental cancer and radiation epidemiology
